# Clinical and Radiographic Results in Thoracic Hyperkyphosis Surgical Treatment Considering the Optimal Distal Fusion Area

**DOI:** 10.3390/jcm13226799

**Published:** 2024-11-12

**Authors:** Laura Scaramuzzo, Fabrizio Giudici, Calogero Velluto, Giuseppe Barone, Maria Concetta Meluzio, Antonino Zagra, Luca Proietti

**Affiliations:** 1 Department of Aging, Orthopaedic and Rheumatological Sciences, Fondazione Policlinico Universitario Agostino Gemelli IRCCS, Largo A. Gemelli, 8, 00168 Rome, Italy; scaramuzzolaura@gmail.com (L.S.); maricomeluzio@gmail.com (M.C.M.); luca.proietti@policlinicogemelli.it (L.P.); 2 Spine Surgery Division I, IRCCS Ospedale Galeazzi—Sant’Ambrogio, 20157 Milan, Italy; giudici.fabrizio61@gmail.com (F.G.); giuseppebarone.gale@gmail.com (G.B.);

**Keywords:** thoracic hyperkyphosis, junctional kyphosis, Scheuermann, fusion area, sagittal stable vertebra

## Abstract

**Introduction:** The aim of this retrospective study was to evaluate the clinical and radiographic outcomes of patients who underwent posterior correction and fusion for thoracic hyperkyphosis, with a focus on determining the optimal distal fusion level. **Methods:** From 2006 to 2012, 26 patients with a median age of 16.8 years (range 14–25), diagnosed with either idiopathic hyperkyphosis or Scheuermann’s kyphosis, underwent posterior fusion and Ponte osteotomies at two medical centers. Radiographic evaluations were performed preoperatively, immediately postoperatively, and at the final follow-up and included assessments of fusion extension, Cobb angle, sagittal balance, and the presence of junctional kyphosis or discopathy. **Results:** The median follow-up period was 12.3 years (range 11–17). Good clinical outcomes were observed in 24 patients, with no cases of hardware failure. The distal fusion area included the first lordotic vertebra in 17 patients, the sagittal stable vertebra in four patients, and both in five patients. Cobb angle correction was maintained at 50% at the final follow-up (*p* > 0.05). Significant sagittal balance correction was achieved in 87% of patients immediately postoperatively and was maintained at the final follow-up (*p* > 0.05). Junctional kyphosis occurred in two patients whose fusion area included only the first lordotic vertebra. **Conclusions:** Including at least the first lordotic vertebra in the fusion area is crucial for preventing junctional kyphosis. Extending the fusion to the stable vertebra can reduce the incidence of distal junctional kyphosis, especially in symptomatic young adult patients, potentially avoiding the need for revision surgery.

## 1. Introduction

The selection of the fusion area in spinal deformity surgery, particularly for the treatment of hyperkyphosis, remains a challenging aspect of clinical practice. While numerous studies have provided clear guidelines for scoliosis surgery, similar guidelines for hyperkyphosis are lacking. Hyperkyphosis includes both Scheuermann’s disease, with an incidence of 1% to 8% in the general population, and idiopathic thoracic hyperkyphosis, which is often mistaken for poor posture [1,2,3,4,5]. Surgical intervention is typically indicated for progressive kyphosis exceeding 65°, deformity progression despite bracing, intractable pain, or unacceptable cosmetic deformity [6,7,8]. Historically, a combined anterior release and posterior instrumentation with fusion was the standard treatment until the Ponte procedure was introduced in 1984. This technique, which involves posterior column shortening with multiple osteotomies, has since popularized posterior-only treatment [9]. Recent studies have emphasized the effectiveness of posterior-only approaches, demonstrating improvements in patient outcomes with minimally invasive techniques [10].

Despite these advancements in surgical techniques, the treatment of thoracic hyperkyphosis remains associated with complications, particularly a significant risk of junctional kyphosis at both the proximal and distal levels. This risk highlights the importance of carefully selecting the fusion area. Established concepts recommend extending the fusion over the kyphotic deformity and using the proximal end vertebra as the upper fusion limit to prevent proximal junctional kyphosis (PJK). However, the criteria for selecting the distal fusion limit remain controversial. Recent studies suggest that stopping the fusion at the end vertebra may be insufficient. Instead, extending the fusion to the first lordotic vertebra (FLV), defined as the vertebra immediately below the first lordotic lumbar disk, is recommended [11,12,13]. Additionally, the Lenke group’s concept of the sagittal stable vertebra (SSV)—the most proximal lumbar vertebra intersected by the posterior sacral vertical line (PSVL)—provides further guidance for determining the distal fusion limit [14,15,16].

This study retrospectively evaluates the clinical and radiographic outcomes of patients undergoing posterior correction and fusion for thoracic hyperkyphosis, with a focus on identifying the appropriate distal fusion level. We hypothesize that including both the first lordotic vertebra and the sagittal stable vertebra in the fusion area will reduce the incidence of distal junctional kyphosis.

## 2. Material and Methods

### 2.1. Study Design

This retrospective study reviewed the clinical and radiographic outcomes of 26 patients (13 males and 13 females) with idiopathic hyperkyphosis and Scheuermann’s kyphosis who underwent surgical treatment between 2006 and 2012 at two medical centers. The mean age of the patients was 16.8 years (range 14–25), with an average follow-up period of 12.3 years (range 11–17). The cohort included 17 patients diagnosed with Scheuermann’s kyphosis and 9 with idiopathic hyperkyphosis [4].

### 2.2. Surgical Procedure

All patients underwent posterior fusion with pedicle screw fixation and Ponte osteotomies. Curve correction was achieved using a combination of cantilever maneuvers with prebent rods and segmental compression at each vertebral space, beginning from the proximal end of the curve [9]. Based on the fusion area, patients were divided into two groups: Group 1, where the fusion ended at the first lordotic vertebra (FLV), and Group 2, where the fusion area included the sagittal stable vertebra (SSV).

### 2.3. Radiographic Assessment

Radiographic evaluations were performed preoperatively, immediately postoperatively, and at the final follow-up using posteroanterior and lateral X-rays. The parameters assessed included thoracic kyphosis (Cobb angle), lumbar lordosis, pelvic incidence, sagittal balance (defined as the horizontal distance between the C7 plumb line and the posterosuperior corner of the sacrum, or SVA), and the presence of junctional kyphosis or discopathy at the distal end, which was evaluated by changes in the distal junctional angle. Additionally, the correlation between pelvic incidence and correction loss at the final follow-up was assessed for all patients and across both groups.

**Figure 1:**
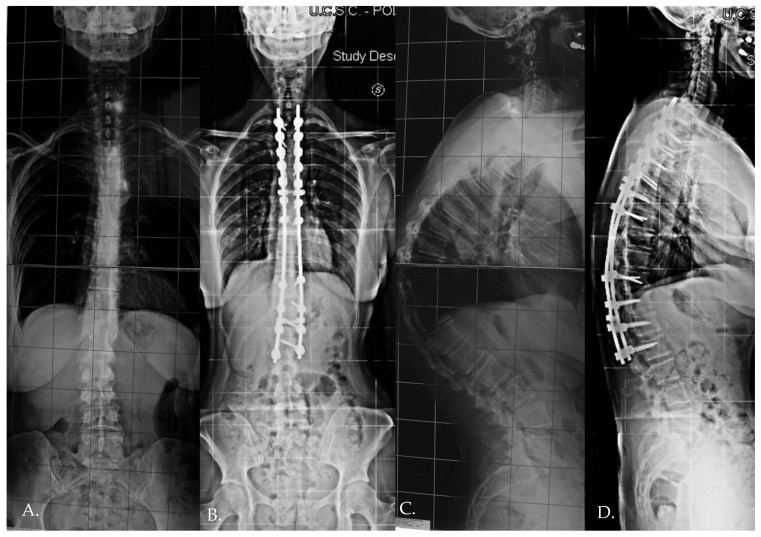
Standing preoperative (**A**,**C**) X-rays and immediate postoperative X-rays (**B**,**D**) of a 23 years old female patient.

The specific measurement methods for assessing these parameters were depicted in detailed radiographic images (Figure 1), which included examples illustrating how the Cobb angle, lumbar lordosis, pelvic incidence, and sagittal balance were measured.

### 2.4. Statistics

Statistical analysis was performed using SPSS software 30.0.0 (SPSS Inc., Chicago, IL, USA). Continuous variables were analyzed using the Mann–Whitney U test due to the small sample size and non-normal distribution of the data. Categorical variables were evaluated using the chi-squared test and, where applicable, Fisher’s exact test was used to confirm results. The significance level was set at *p* < 0.05. Additionally, Cohen’s d effect sizes were calculated to assess the magnitude of differences in reported measurements at each time point. A sample size estimate was performed using power analysis to determine if the sample size was adequate to support the study’s claims [17,18,19]. The sample size was estimated using power analysis to ensure it was adequate to detect statistically significant differences in clinical outcomes. The calculations were based on anticipated effect sizes and variability in the primary outcome measures. The post hoc sample size analysis revealed that a minimum of 41 patients per group (total of 82 patients) would be required to detect a statistically significant difference with a power of 80% and a significance level (α) of 0.05. Our study included 26 patients, which represents a limitation due to the smaller sample size. However, considering the rarity of this pathology and the infrequency of such surgical procedures, our sample size is still valuable and provides meaningful insights into this complex clinical condition.

## 3. Results

The median follow-up period was 12.3 years (range 11–17 years). The average surgical time was 240 min (range 180–300 min), and the average intraoperative blood loss was 400 mL (range 250–850 mL). The median length of hospital stay after surgery was 8 days (range 7–10 days), with all patients successfully mobilized by the second postoperative day. Three patients were lost to follow-up. Group 1 (fusion ending at the FLV) included seven patients with a mean age of 20.2 years (SD 6.3), while Group 2 (fusion including the SSV) included 16 patients with a mean age of 23.5 years (SD 4.2). The demographic data are summarized in Table 1.

The mean preoperative thoracic kyphosis was 77.2° (SD 10.1), which decreased to 43.7° (SD 8.5) immediately postoperatively (*p* < 0.001). The overall mean correction loss was 2.6° (*p* > 0.05), while Group 1 had a mean correction loss of 3.6°, and Group 2 had a mean correction loss of 2.2°, as summarized in Table 2. At the final follow-up, the thoracic kyphosis measured 46.3° (SD 9.2), with a non-significant mean correction loss of 2.6° (*p* > 0.05), as summarized in Table 2.

Changes in lumbar lordosis were also analyzed. The mean preoperative value was 63.2° (SD 10.2), which decreased to 44.9° (SD 9.7) immediately postoperatively (*p* = 0.001). At the final follow-up, the mean correction loss was 1.9° (*p* > 0.05), as shown in Table 3.

The sagittal balance, measured as the horizontal distance between the C7 plumb line and the posterosuperior corner of the sacrum (SVA), changed from −0.3 cm (range: −8.4 to 3.6 cm) preoperatively to −0.2 cm (range: −2.9 to 6.4 cm) immediately postoperatively. Further correction was observed at the final follow-up, with a value of −0.8 cm (range: −3.4 to 3.0 cm) (*p* = 0.08). Distal junctional kyphosis (DJK) occurred in two patients, both in Group 1, where the fusion area included only the first lordotic vertebra (Table 4). One patient remained asymptomatic and maintained satisfactory correction at the 3-year follow-up, while the other developed significant symptoms that required revision surgery.

The mean pelvic incidence was 44.8° (range 30–65°), and no correlation between the preoperative pelvic incidence and postoperative correction loss was observed in our series. For all patients, the mean preoperative thoracic kyphosis was 77.2° (range 64–98°), which decreased to 43.7° (range 32–63°) immediately postoperatively (*p* = 0.001). A non-statistically significant correction loss of 2.6° (*p* > 0.05) was observed at the final follow-up, with a mean thoracic kyphosis of 46.3° (range 35–78°). A similar trend was observed when the two groups were analyzed separately (Table 5 and Table 6). The correction rate was similar in both groups, and while loss of correction was greater in Group 1 than in Group 2, this difference was not statistically significant (*p* = 0.30).

When considering the two groups separately, the correction rate was better in Group 1 (*p* = 0.04), while the loss of correction was similar between both groups (*p* > 0.05) (Table 5 and Table 6). The SVA changed from −0.3 cm (range: −8.4 to 3.6 cm) preoperatively to −0.2 cm (range: −2.9 to 6.4 cm) immediately postoperatively. Further correction was observed at the final follow-up, with a value of −0.8 cm (range: −3.4 to 3 cm), though this was not statistically significant (*p* = 0.08).

When analyzing only Group 1, the SVA changed from −1.2 cm (range: −3.9 to 0.2 cm) preoperatively to −0.4 cm (range: −2.8 to 2.1 cm) immediately postoperatively (*p* = 0.05). A further correction loss was seen at the final follow-up, with a value of −1.4 cm (range: −1.5 to 3 cm), which was statistically significant (*p* = 0.04). A similar trend with better correction maintenance was observed in Group 2, where the SVA changed from 0.2 cm (range: −8.4 to 4.6 cm) preoperatively to −0.2 cm (range: −2.9 to 6.4 cm) immediately postoperatively (*p* = 0.03). However, at the final follow-up, a non-statistically significant correction loss was observed, with a value of −0.9 cm (range: −3.4 to 1.3 cm) (*p* = 0.40). Changes in the distal junctional angle (DJA) were also evaluated. For all patients, the mean preoperative DJA was 7.0° (range: 2.2–15.1°), which decreased to 4.5° (range: −7.5 to 8.5°) immediately postoperatively (*p* = 0.02). A correction loss of 1° was seen at the final follow-up, with a mean DJA of 3.5° (range: −8.3 to 0.1°).

When analyzing the two groups:

- In Group 1, the mean preoperative DJA was 4.8° (range: 2.2–7.4°), which decreased to 2.2° (range: −7.5° to 6.1°) immediately postoperatively (*p* = 0.005). At the final follow-up, the DJA was 0.7° (range: −8.3° to 6.1°).
- In Group 2, the mean preoperative DJA was 7.9° (range: 0.2–8.4°), which decreased to 5.6° (range: 0.2–8.5°) immediately postoperatively (*p* = 0.009). At the final follow-up, the DJA was 4.9° (range: 0.2–7.1°), with a non-statistically significant correction loss of 0.7°.

**Figure 2:**
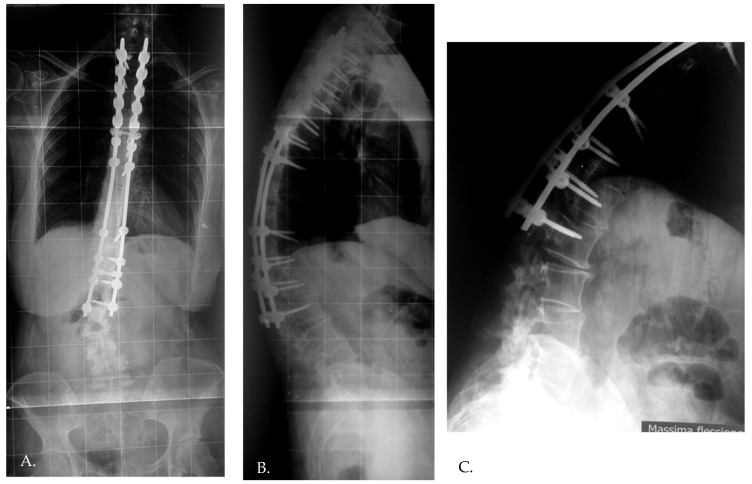
The development of distal junctional kyphosis and implant failure reported at the 3 year follow-up (**A**–**C**).

**Figure 3:**
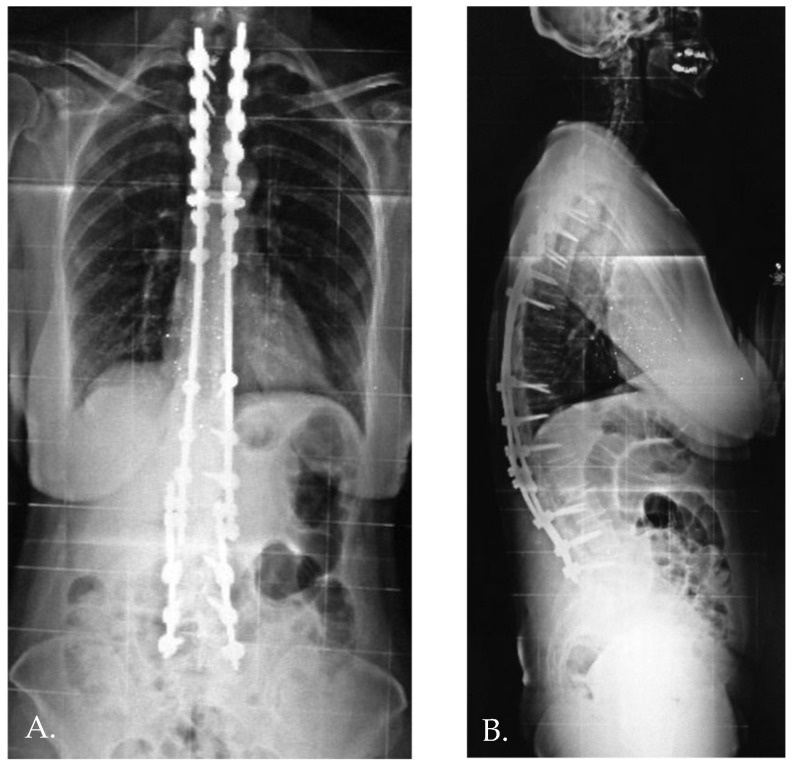
Anteroposterior and standing lateral radiographs (**A**,**B**) after revision surgery.

Distal junctional kyphosis (DJK) occurred in two patients, both in Group 1, where the fusion area included only the first lordotic vertebra. One patient, a 24-year-old female, was asymptomatic and maintained satisfactory correction at the 3-year follow-up. The second patient, a 23-year-old female, developed significant symptoms at the 3-year follow-up, including distal implant failure (Figure 2), which worsened her clinical condition. She experienced intractable back pain and significant sagittal and coronal imbalance. Due to hardware failure and clinical deterioration, revision surgery was required. The revision included extending the fusion to L5, interbody fusion between L1 and L2, and multiple Smith–Petersen osteotomies (Figure 3). At the 6-month follow-up, the patient showed good balance and a satisfactory reduction in pain.

## 4. Discussion

Junctional kyphosis (JK) is a significant complication in the surgical treatment of hyperkyphosis, with incidence rates varying widely in the literature. Multiple studies have identified various causes for JK, including overcorrection of the curve, choice of instrumentation, improper selection of the fusion area, and specific surgical techniques such as cantilever reduction or segmental compression techniques [12]. In our series, cantilever maneuvers combined with segmental compression aimed to distribute reduction forces evenly, thereby mitigating the risk of JK. In our study, the overall incidence of distal junctional kyphosis (DJK) was 8%, with a significantly higher incidence (28%) observed in Group 1 (fusion ending at the first lordotic vertebra, FLV). This finding is consistent with previous reports by Lowe and Kasten [14] and Denis [12], which indicate that terminating fusion short of the first lordotic vertebra significantly increases the risk of DJK. These studies demonstrated that a lordotic disk is less prone to local kyphosis, and the vertebra below the first lordotic disk often serves as a stable vertebra in the sagittal plane, which is crucial for maintaining proper spinal alignment.

The Lenke group’s introduction of the sagittal stable vertebra (SSV) concept has further provided insights into reducing the risk of JK. They suggested that the distal limit of the fusion should include the SSV to ensure the fusion mass is centered over the sacrum, thereby enhancing stability and reducing the risk of negative sagittal balance and subsequent JK [11]. In our study, patients in Group 1 exhibited more negative sagittal balance than those in Group 2, although this difference was not statistically significant. Sagittal balance is influenced by complex biomechanical mechanisms. Patients with thoracic hyperkyphosis often exhibit increased lumbar lordosis as a compensatory mechanism to preserve overall balance. Jansen et al. [16] demonstrated that surgical correction of thoracic hyperkyphosis typically results in decreased lumbar lordosis, primarily in the upper lumbar spine. DJK may represent both a progression of pathological thoracic kyphosis and a postoperative compensatory mechanism to prevent the loss of lumbar lordosis and maintain sagittal balance. Extending the fusion to the stable vertebra minimizes this compensatory mechanism, subsequently reducing the incidence of DJK.

Contrary to some reports [20,21], our study found no association between the risk of DJK and parameters such as the amount of correction or preoperative curve magnitude [13]. However, in our experience, the amount of correction can become a significant risk factor, particularly in adults as reported in Literature [22,23]. This is illustrated by the case of a symptomatic patient in our series, where excessive correction (from 98° to 32°) combined with a fusion that ended short of the SSV (initially thought to be L2, but later identified as L3) led to implant failure and revision surgery. The lumbar lordosis correction in this patient was inadequate to maintain correction over time, underscoring the importance of accurate spinopelvic alignment in preventing JK [24].

The clinical significance of DJK varies, with several studies reporting a prevalence of asymptomatic cases [12,25,26]. Mild DJK often goes unnoticed without detailed postoperative examinations, highlighting the need for regular and thorough postoperative evaluations to prevent severe spinal imbalance. Regular follow-up is crucial to identify and address DJK early, even in asymptomatic patients, to avoid long-term complications and the potential need for revision surgery [27,28,29]. Recent studies have shown that advanced surgical techniques, such as the “kickstand rod” technique, can be beneficial in correcting severe coronal and sagittal malalignments in adult spinal deformity, providing stable radiological and functional outcomes [30]. This technique has shown promise in managing complex spinal deformities, which are particularly challenging in hyperkyphosis cases [31]. Additionally, understanding the epidemiology, diagnosis, and management of conditions like Baastrup’s disease is essential for comprehensive patient care and for preventing complications post-surgery [32]. Baastrup’s disease, characterized by close approximation and contact of adjacent spinous processes, often coexists with spinal deformities like hyperkyphosis, and its management is crucial for improving overall surgical outcomes.

Our study’s findings also underscore the importance of considering individual patient anatomy and biomechanics when planning the extent of surgical fusion. The variability in pelvic incidence and corresponding sagittal alignment parameters necessitates a personalized approach to each patient’s surgical plan. This consideration is particularly crucial in adolescent patients, whose growth and development can further impact the long-term outcomes of spinal fusion surgery.

### Limitations

This study has several limitations. The small sample size limits the generalizability of our findings, although it is comparable to other published series on this topic. Additionally, the retrospective nature of the study presents inherent biases, including selection bias and the potential for incomplete data. Despite these limitations, the uniform surgical approach and instrumentation used in our series (bilateral segmental pedicle screw fixation) enhance the comparability of our data by eliminating instrumentation bias, which is a common confounding factor in multicenter studies. Another limitation is the lack of a standardized follow-up protocol, which could have resulted in variations in the timing and frequency of postoperative evaluations. Furthermore, the reliance on radiographic measurements alone, without correlating clinical outcomes, limits the ability to fully understand the impact of DJK on patient quality of life. Future studies should aim to include standardized clinical outcome measures alongside radiographic evaluations to provide a more comprehensive assessment of surgical success and complications. Future research should focus on larger, prospective studies to further validate these findings and provide more definitive guidelines for the optimal selection of fusion levels in hyperkyphosis surgery. Additionally, incorporating advanced imaging techniques and biomechanical modeling could enhance the understanding of spinal alignment and its impact on long-term outcomes. Collaborative efforts across multiple centers could also help gather a larger dataset, providing more robust evidence to guide clinical practice.

## 5. Conclusions

The selection of fusion levels in the surgical treatment of hyperkyphosis is critical in preventing junctional complications. Including at least the first lordotic vertebra in the fusion area is essential to reduce the incidence of distal junctional kyphosis (DJK). Extending the fusion to the sagittal stable vertebra (SSV) further decreases the risk, particularly in symptomatic young adult patients, and can potentially avoid the need for revision surgery. Our findings support the use of the SSV as the distal fusion landmark to achieve optimal outcomes in hyperkyphosis correction.

## Figures and Tables

**Table 1:** Patient demographics.

Parameter	Group 1 (*n* = 7)	Group 2 (*n* = 16)	*p*-Value
Age (years)	20.2 ± 6.3	23.5 ± 4.2	0.12
Gender (Male/Female)	4/3	9/7	0.85
Follow-up period (years)	12.1 ± 1.5	12.4 ± 1.3	0.67
Pelvic incidence (degrees)	44.8 ± 8.7	45.2 ± 9.1	0.92

**Table 2:** Thoracic kyphosis.

Measurement	Group 1 (*n* = 7)	Group 2 (*n* = 16)	*p*-Value	Cohen’s d
Preoperative (degrees)	81.4 ± 9.5	75.4 ± 10.3	0.18	-
Immediate post-op (degrees)	43.3 ± 7.8	43.9 ± 9.0	<0.001	-
Final follow-up (degrees)	46.9 ± 8.2	46.1 ± 9.7	0.77	-
Correction loss (degrees)	3.6 ± 1.2	2.2 ± 1.0	0.30	-
Pre–post effect Size	-	-	-	3.89 (Group 1), 2.93 (Group 2)

**Table 3:** Lumbar lordosis.

Measurement	Group 1 (*n* = 7)	Group 2 (*n* = 16)	*p*-Value	Cohen’s d
Preoperative (degrees)	60.7 ± 10.1	64.3 ± 10.4	0.45	-
Immediate post-op (degrees)	38.9 ± 8.5	47.5 ± 10.1	0.14	-
Final follow-up (degrees)	41.3 ± 9.2	49.6 ± 10.0	0.12	-
Correction loss (degrees)	2.4 ± 1.0	1.7 ± 0.8	0.45	-
Pre–post effect size	-	-	-	2.20 (Group 1), 1.50 (Group 2)

**Table 4:** Distal junctional kyphosis.

Parameter	Group 1 (*n* = 7)	Group 2 (*n* = 16)	*p*-Value	Cohen’s d
DJK occurrence (n)	2	0	0.04	-
Symptomatic DJK (n)	1	0	0.12	-
Revision surgery (n)	1	0	0.12	-
Pre–post effect size	-	-	-	1.75 (Group 1), 0.85 (Group 2)

**Table 5:** Summary of preoperative and postoperative data of idiopathic and Scheuermann hyperkyphosis group 1: fusion to FLV.

Group 1: FLV = 7	Preop	Postop	Last
Thoracic Kyphosis	81.4	43.3	46.9
Correction amount			34.5
Correction lost			3.6
LL	60.7	38.9	41.3
Correction amount			19.4
PI	41.8	41.8	41.8
Disk junctional angle	4.8	2.2	0.7
Correction lost			4.1
SVA	−1.2	−0.4	−1.4
Fused vertebra			10.4
DJK			2

Abbreviations’ legend: FLV = first lordotic vertebra; PI = pelvic incidence; LL = lumbar lordosis; DJK = distal junctional kyphosis; SVA = sagittal vertical axis line.

**Table 6:** Summary of preoperative and postoperative data of idiopathic and Scheuermann hyperkyphosis group 2: fusion to SSV.

Group 2: SSV = 16	Preop	Postop	Last
Thoracic Kyphosis	75.4	43.9	46.1
Correction amount			29.3
Correction lost			2.2
LL	64.3	47.5	49.6
Correction amount			14.7
PI	42.6	42.6	42.6
Disk junctional angle	7.9	5.6	4.9
Correction lost			3.0
SVA	0.2	−0.2	−0.9
Fused vertebra			11.2
DJK			NO

Abbreviations’ legend: SSV = sagittal stable vertebra; PI = pelvic incidence; LL = lumbar lordosis; DJK = distal junctional kyphosis; SVA = sagittal vertical axis line.

## Data Availability

Data available on request from the authors.
